# Musculoskeletal congenital malformations: do paternal occupational exposures play a role?

**DOI:** 10.1007/s11832-014-0594-z

**Published:** 2014-05-25

**Authors:** Ayman M. Ali, Mohamed Abdelaziz, Barakat El-Alfy

**Affiliations:** Mansoura University, Mansoura, Daqahlia 35516 Egypt

**Keywords:** Paternal, Occupational exposures, Musculoskeletal congenital malformations

## Abstract

**Background:**

Paternal occupational exposures to potential health hazards are likely to affect congenital malformations through the spermatogenesis cycle.

**Purpose:**

The aim of this case–control study was to assess the relationship between the risk of musculoskeletal congenital malformations in offspring and paternal workplace exposure to potential health hazards during the preconception period.

**Method:**

The study comprised 105 patients (cases) with a musculoskeletal congenital malformation(s) and 135 controls matched for age and demographic characteristics. Both parents of each case and control were interviewed in the hospital by a trained physician. They also completed a questionnaire focusing on the preconception period and on the 3-month period immediately before and after the pregnancy conception date, respectively, of the child under study.

**Results:**

The odds of having a child with a congenital malformation was higher (*P* < 0.05) if the father was occupationally exposed to pesticides, solvents, or welding fumes during the preconception period.

**Conclusion:**

Control of workplace exposures and adherence to threshold limit values of these potential health hazards should be adopted to minimize the risk of fathers having offspring with a congenital malformation.

## Introduction

The cause of a disruption in reproduction may be the result of genetic and/or physiological events that occur in the mother, father and/or child [1]. In animal studies, paternal exposure to numerous agents, including environmental chemicals, recreational substances, and therapeutic drugs, has been shown to negatively affect reproductive success, resulting in adverse reproductive outcomes, including congenital malformations [2].

Historically, studies assessing the role of occupational exposure as etiological agents for birth defects have focused on maternal exposures during pregnancy [2]. The role of paternal exposure has received less attention despite evidence from animal models showing that exposures of males to toxic agents may result in congenital malformations in future offspring [3–6].

Th objective of this study was, therefore, to investigate the association between the occurrence of musculoskeletal congenital malformations in offspring and the paternal work-place exposures.

## Methods

This case–control study was conducted between 2011 and 2013. All participants signed a written formal consent form to participate in the study after being informed of the study objectives. A case patient was defined as a child diagnosed with any type of musculoskeletal congenital malformation, as were classified by the International Classification of Diseases, tenth revision (ICD-10) [7].

### Sample

During the period from May 2011 to January 2013, all musculoskeletal congenital malformation cases were recruited during their initial hospitalization for surgery in our institution. Cases were excluded if one or more of the following conditions were present: (1) one or both parents were dead or not present at the time of interview; (2) the mother had history of disease or drug intake during pregnancy; (3) the father and/or mother had a history of diabetes mellitus (DM), renal or liver diseases; (4) there was a family history of genetic diseases or congenital malformations. In addition, any case with a paternal history of medical problems related to the reproductive or endocrine system was excluded from the study. The total number of recruited cases was 155. However, due to parental refusal to participate (21 cases), maternal history of infections or drug intake during pregnancy (17 cases), family history of congenital malformations (5 cases), and history of paternal DM (7 cases), the final sample size was 105 cases.

A control group of 135 children without birth defect, cancer, or genetic disease was selected by simple random sampling among children who had been admitted for the treatment of some other disorder, most often infections or a need for minor surgery (fractures). Children in the control group were matched to the cases for age and demographic characteristics.

### Data collection

Both parents, where possible, of the cases and controls were interviewed in the hospital by a trained physician; they also completed a questionnaire which focused on the preconception period and on the 3-month period immediately before and after the pregnancy conception date, respectively, of the child under study. The questionnaire included questions on personal history, socioeconomic features, smoking, residence, past history of diseases, family history of congenital malformations, history of consanguinity, mother’s medical and obstetrical history, mother’s exposure to X rays and surgery during pregnancy, as well as infectious diseases, and drug intake, infection of the reproductive system, metabolic disorders, and endocrine abnormality. It also included questions on the father’s and mother’s occupation according to the International Standard Classifications of Occupations (ISCO-08) [8], as well as paternal and maternal occupational exposure to pesticides, solvents (including glues, adhesives, polishes, thinner or turpentine), welding fumes, and lead, as well as working with video display terminals (VDTs) and computer monitors. “Yes” was considered to be the correct answer for occupational exposure if the exposure was frequent and occurred during the peri-conceptional period of the pregnancy of the respective child.

### Analysis

The first step in our analysis was to compare the baseline demographic data between the cases and controls. Our second step was to perform bivariate analyses to determine the association between congenital malformation(s) and exposure factors based on a priori hypotheses. These bivariate analyses were performed using Student’s *t* test for continuous variables and the Pearson chi-squared and Fisher’s exact tests for categorical and dichotomous variables. Next, we employed a multivariable logistic regression model using the forward-looking Wald strategy. Candidate variables were considered to have a bivariate association with congenital malformations at *P* < 0.05. The odds ratio and 95 % confidence intervals were calculated for case–control associations with factors suspected to be associated to congenital malformations, with adjustments for confounders (including ages of father and mother at child’s birth, maternal occupation(s), child’s age and sex, paternal and maternal education, family residence and income, paternal smoking, consanguinity). Statistical analysis was performed using SPSS ver. 16.0 (SPSS Inc., Chicago, IL) on a personal computer. A two-tailed *P* value of <0.05 was considered to be statistically significant.

## Results

Among all cases of congenital malformations (*N* = 105), lower limb deformity was the most frequent (38.3 %), followed by upper limb deformity (34.4 %), vertebral malformations (14.4 %), and malformation of the clavicle and shoulder region (5.9 %). Craniofacial and thoracic skeleton malformations were the least frequent (3.1 and 0.8 %, respectively). There were three cases (3 %) of multiple anomalies (Table 1).

**Table 1:** Prevalence of congenital malformations among cases (*N* = 105)

Congenital malformation	Number of cases
Tibial pseudoarthrosis	4
Hallux varus	3
Talipes equinovarus	21
Congenital hip dislocation	10
Congenital short lower limb	2
Syndactyly	9
Polydactyly	4
Cleft hand	4
Madelung’s deformity	3
Club hand	4
Absent thumb	1
Clinodactyly	2
Bifid thumb	1
Radioulnar synostosis	3
Macrodactyly	3
Phocomelia	2
Spina bifida	2
Fused vertebrae	2
Sacral agenesis	3
Congenital scoliosis	4
Spondylolisthesis	4
Congenital elevation of scapula	3
Congenital glenoid hypoplasia	1
Pseudoarthrosis of the clavicle	2
Craniosynostosis	1
Cleft palate	2
Bifid rib	1
Pectus carinatum	1
Multiple anomalies	3

The cases did not differ significantly from the controls in terms of paternal and maternal age at child’s birth, child’s sex, and child’s age at the interview. There was also no statistical significant difference between the cases and controls for paternal and maternal educational levels, family residence, family income, and paternal smoking during the periconceptional period. There was also no reported significant difference in maternal smoking between both groups (Table 2).

**Table 2:** Basic demographic characteristics of the cases and controls

Demographic characteristics	Cases (*N* = 105)	Controls (*N* = 135)	Test	*P*
Paternal age at birth of child (years)	33.9 ± 7.1	34.24 ± 6.9	0.15	0.71
Maternal age at birth of child (years)	27.9 ± 6.1	28.1 ± 6.0	0.45	0.59
Child’s age at the interview (years)	3.2 ± 2.8	2.9 ± 2.8	0.7	0.43
Child’s sex				
Male	70 (66.7)	50 (73.7)	2.7	0.05
Female	35 (33.3)	85 (26.3)		
Paternal educational level				
Illiterate	14 (13.3)	19 (14.1)	0.1	0.96
Read and write/primary school	15 (14.3)	20 (14.8)		
Preparatory/secondary school	41 (39.1)	52 (38.5)		
University graduate or higher	35 (33.3)	44 (32.6)		
Maternal educational level				
Illiterate	11 (10.5)	15 (10.7)	4.3	0.18
Read and write/primary school	19 (18.1)	23 (17.4)		
Preparatory/secondary school	51 (48.5)	64 (47.0)		
University graduate or higher	24 (22.9)	33 (24.8)		
Family residence				
Rural	77 (73.3)	102 (75.6)	0.4	0.29
Urban	28 (26.7)	33 (24.4)		
Family income				
Sufficient	45 (42.9)	64 (47.8)	1.5	0.22
Not sufficient	60 (57.1)	71 (52.2)		
Paternal smoking				
Non-smoker	43 (41)	65 (48.1)	2.6	0.19
Current smoker	62 (59)	70 (51.9)		

Data are presented as the mean ± standard deviation or as the number of cases or controls, with the percentage in parenthesis

The most prevalent paternal occupations among the cases were those of craftsmen and related trades(men) and skilled agricultural and fishery workers (28.6 and 19.04 %, respectively). These working groups were significantly more frequent among the cases than among the controls (17.7 and 8.9 %, respectively). Professionals and clerks were less frequent among the cases than among the controls, and the difference was statistically significant. In terms of maternal occupations, no statistical significant differences were observed between cases and the controls, and most of the mothers of both groups were non-employed (84.8 and 76.3.9 %, respectively) (Table 3).

**Table 3:** Paternal and maternal occupations among the cases and controls

Occupations	Cases (*N* = 105)	Controls (*N* = 135)	Test	*P*
Paternal occupations				
Professionals	14 (16.5)	32 (24.1)	4.1	0.01
Technicians and associate professionals	16 (12.4)	18 (13.3)	0.1	0.42
Clerks	7 (10.7)	26 (19.3)	7.1	0.00
Service workers and shop and market sales workers	4 (3.7)	7 (5.2)	0.6	0.54
Skilled agricultural and fishery workers	20 (17.8)	12 (8.9)	8.6	0.00
Craft and related trades workers	30 (26.0)	24 (17.7)	5.2	0.05
Plant and machine operators and assemblers	11 (10.3)	10 (7.0)	1.7	0.19
Elementary occupations	2 (1.2)	5 (4.1)	3.9	0.07
Armed forces	1 (0.8)	1 (1.1)	0.1	0.98
Maternal occupations				
Non-employed	89 (84.8)	103 (84.4)	6.84	0.15
Professionals	11 (10.5)	12 (7.8)		
Clerks	1 (0.9)	5 (2.2)		
Service workers and shop and market sales workers	1 (0.9)	0 (0.0)		
Skilled agricultural and fishery workers	3 (2.9)	15 (5.6)		

Data are presented as the number of cases or controls, with the percentage in parenthesis

Paternal work-place exposure to pesticides (21.9 %), solvents (20.9 %), welding fumes (16.2 %), and lead (11.4 %) were more frequent among the cases than among the controls (8.15, 4.4, 3.7, and 2.2 %, respectively), and the differences were highly statistically significant differences. However, paternal workplace exposure to VDTs and computers did not differ between the cases (11.4 %) and the controls (8.9 %) (Table 4).

**Table 4:** Paternal workplace exposures during periconceptional period among the cases and controls

Paternal workplace exposures^a^	Cases (*N* = 105)	Controls (*N* = 135)	*χ* ^2^	*P*
Pesticides	23 (21.9)	11 (8.15)	14.3	0.00
Solvents	22 (20.9)	6 (4.4)	27.1	0.00
Welding fumes	17 (16.2)	5 (3.7)	21.1	0.00
Lead	12 (11.4)	3 (2.2)	14.6	0.00
VDT and computers	12 (11.4)	12 (8.9)	0.4	0.65

Data are presented as the number of cases or controls, with the percentage in parenthesis

^a^Multiple exposures constituted 9 % of the total reported exposures among cases and 5.5 % among the controls. Moreover, 25.6 % of cases and 73.0 % of the controls reported no exposures to the studied exposure factors

Paternal workplace exposures to pesticides, solvents, and welding fumes were significantly associated with an increased risk of congenital malformations among offspring. Exposure to lead was not significantly associated with increased risk of congenital malformations (Table 5).

**Table 5:** Odds ratios of the risk factors associated with congenital malformations among the study population

Risk factors	*B*	Odds ratio	95 % Confidence interval	*P*
Workplace exposures^a^				
Pesticides	1.21	3.40	(1.94–5.88)	0.00
Solvents	1.76	5.69	(2.88–11.52)	0.00
Welding fumes	1.02	2.80	(1.19–7.28)	0.03
Lead	1.09	2.99	(0.99–8.64)	0.07
Consanguinity	0.61	1.88	(1.21–2.87)	0.00

^a^Chi-square of the logistic regression model = 79.52 (*P* = 0.00); corrected percentage = 67.0 %

## Discussion

Knowledge of the etiology of congenital defects is important not only for a thorough understanding of their genesis, but also for the prevention of these disorders through eliminating or decreasing exposures to environmental factors found to be causative [2].

Our study was designed to analyze the relationship between paternal occupational exposures and the increased probability of having a child with a congenital malformation. We used standard international classifications both for the type of congenital anomaly [7] and for the workplace occupation [8]. Taking into account biological factors and experimental evidence from previous studies [9, 10], we based our occupational analysis on the type of activity carried out during the previous year and during the first trimester of the index gestation, including information on work load, work sector, products, and equipment/machinery most frequently used.

Many epidemiological studies have examined the effects of paternal occupational exposures on offspring. In most such studies carried out to date, paternal occupational/industrial exposure involves exposure to multiple agents, rendering it difficult to identify the causative agent(s) [11]. However, some suggestive evidence for specific associations has been reported, providing directions for future epidemiological studies. An increased incidence of spontaneous abortion or miscarriage has been linked to paternal exposures to anesthetic gases, metals (mercury and lead), solvents, pesticides, and hydrocarbons [9].

Regarding paternal occupations, we found that the fathers of children with congenital malformation(s) (cases in our study) were significantly more likely than the fathers of the controls to be actively employed as craftsmen or in related trades, or to be skilled agricultural and fishery workers; the former were also significantly less likely to be professionals or clerks. There was no significant association between maternal occupation and developing a congenital malformation. These results are in agreement with earlier results showing that fathers employed as janitors, woodworkers, firemen, electrical workers, printers, and painters were at increased risk of having a child with a birth defect [11, 12]. Exposures related to these occupations include solvents, wood and wood products, metals, and pesticides [13]^.^

Our results also demonstrate that the fathers who were occupationally exposed to pesticides were significantly more likely to have a child with a congenital anomaly. This finding is in agreement with the results of Schwartz and Logerfo [14] who found a stronger association between the occurrence of limb reduction defects and parental involvement in agricultural work among infants with additional anomalies compared with infants with only limb reduction defects. This finding is compatible with a malformation syndrome induced by a specific agricultural exposure. Lacasana et al. [15] reported that exposure of the father to pesticides before or during the preconception period can also increase the risk of having an anencephalic child.

In our study, paternal occupational exposure to solvents was significantly associated with congenital malformations. However, the small numbers of cases, especially given the different exposure levels and the self-reported nature of exposure and outcome variables may hamper the interpretation of the results.

Kolstad et al. [16] analyzed semen quality in men exposed to organic solvents and found a decline in sperm density, total sperm count, and the proportion of sperm with normal morphology. Lawson et al. [17] reported that pregnancy outcomes among wives of male chemical workers who had been exposed to high levels of organic solvents reflected an enhanced risk of low birth weight or preterm delivery and birth defects through the paternal route. Munger et al. [18] noted an association between well water contaminated with the herbicide atrazine and excess cardiovascular, urogenital, and limb reduction defects. In a Nebraska study, atrazine in the water supply was also associated with excess syndactyly and limb reduction defects [19].

Lead is known to be excreted in the seminal fluid [20], and there is evidence that lead affects the semen quality of exposed workers. Thus, it is biologically plausible that lead may be associated with reproductive hazards. However, in our study lead was not a significant risk factor of congenital malformations. Sallmen et al. [21] also did not find a connection between paternal lead exposure and any specific type of malformation. Irgens et al. [22] reported that among their study cohort the offspring of fathers exposed to lead had no increased risks of having children with of the birth defects under study; however, they did find that mothers exposed to lead had a significantly increased risk of having children with neural tube defects.

We were unable to relate the risk of having a child with a congenital malformation to paternal exposure to any one specific biological marker as the fathers in our study were exposed to a wide diversity of chemicals. Chia and Shi [2] reported that, in most studies on occupational exposures and congenital malformations, it is difficult to assess the extent of exposures accurately. However, the sample size of our study was sufficiently large and the participation rate was high. Further studies are needed to examine the risk of a congenital malformation and paternal occupational exposures through biological monitoring and specific assessment of exposures.

In conclusion, paternal workplace exposures to pesticides, solvents, and welding fumes may be risk factors for having offspring with a congenital malformation. Thus, the implementation of control measures for limiting exposures at the workplace, adherence to threshold limit values, proper use of personal protective equipment and respirators, and health education on the safety and hazards of occupational exposure to chemicals should be adopted to minimize the risk of workers having offspring with a congenital malformation. A further study is required to evaluate the relationship between the length and level of specific occupational exposure and congenital anomalies.
